# Adaptive deep brain stimulation controls levodopa‐induced side effects in Parkinsonian patients

**DOI:** 10.1002/mds.26953

**Published:** 2017-02-17

**Authors:** Manuela Rosa, Mattia Arlotti, Sara Marceglia, Filippo Cogiamanian, Gianluca Ardolino, Alessio Di Fonzo, Leonardo Lopiano, Emma Scelzo, Aristide Merola, Marco Locatelli, Paolo M. Rampini, Alberto Priori

**Affiliations:** ^1^ Clinical Center for Neurostimulation, Neurotechnology, and Movement DisordersFondazione IRCCS Ca' Granda Ospedale Maggiore PoliclinicoMilanItaly; ^2^ Department of Electronics, Computer Science and SystemsUniversity of BolognaCesenaItaly; ^3^ Department of Engineering and ArchitectureUniversity of TriesteTriesteItaly; ^4^ Unit of Clinical NeurophysiologyFondazione IRCCS Ca' Granda Ospedale Maggiore PoliclinicoMilanItaly; ^5^ Dino Ferrari Center, Neuroscience SectionFondazione IRCCS Ca' Granda Ospedale Maggiore PoliclinicoMilanItaly; ^6^ Department of Neuroscience “Rita Levi Montalcini”University of TurinTurinItaly; ^7^ Unit of Stereotactic Functional and Neuroendoscopic NeurosurgeryFondazione IRCCS Ca' Granda Ospedale Maggiore PoliclinicoMilanItaly; ^8^ Department of Health SciencesUniversity of Milan & Ospedale San PaoloMilanItaly

The potential superior benefits of adaptive deep brain stimulation (aDBS) approaches^1^ compared to classical, constant‐parameters DBS were already proven by scientific evidence from different research groups.^2^, ^3^, ^4^ aDBS provides better symptoms control in Parkinson's disease patients by adapting the stimulation parameters to the patient's clinical state estimated through the analysis of subthalamic neuronal oscillations (ie, local field potentials) in the beta band (13‐30 Hz).^5^

Because aDBS administration was never systematically assessed during prolonged stimulation sessions in more ecologic conditions, we tested unilateral aDBS delivered for 2 hours, with specific focus on the concurrent administration of levodopa treatment, in freely moving parkinsonian patients.

We therefore randomly administered aDBS and cDBS through an external wearable prototype^6^ in 10 PD patients with DBS electrode implant in 2 different experimental sessions taking place the 5th and the 6th day after surgery (Fig. 1A). Each experimental session lasted 2 hours, during which the patient, after a baseline assessment (OFF DBS and OFF medication, stimOFF/medOFF), received both levodopa and stimulation (aDBS or cDBS), thus allowing one to study the interaction between electrical and pharmacological stimulation (ON DBS and ON medication, stimON/medON). The patient was blind to the type of DBS received during the session. The clinical effects were blindly evaluated through the UPDRS III (motor part) and the Unified Dyskinesia Rating Scale (UDysRS). According to the gold standard, the clinical assessment was performed by a blinded video rater (rigidity scores were excluded from the analysis). The total electrical energy delivered (TEED) was used for energy efficiency assessment and adverse events were collected for safety assessment.

**Figure 1:**
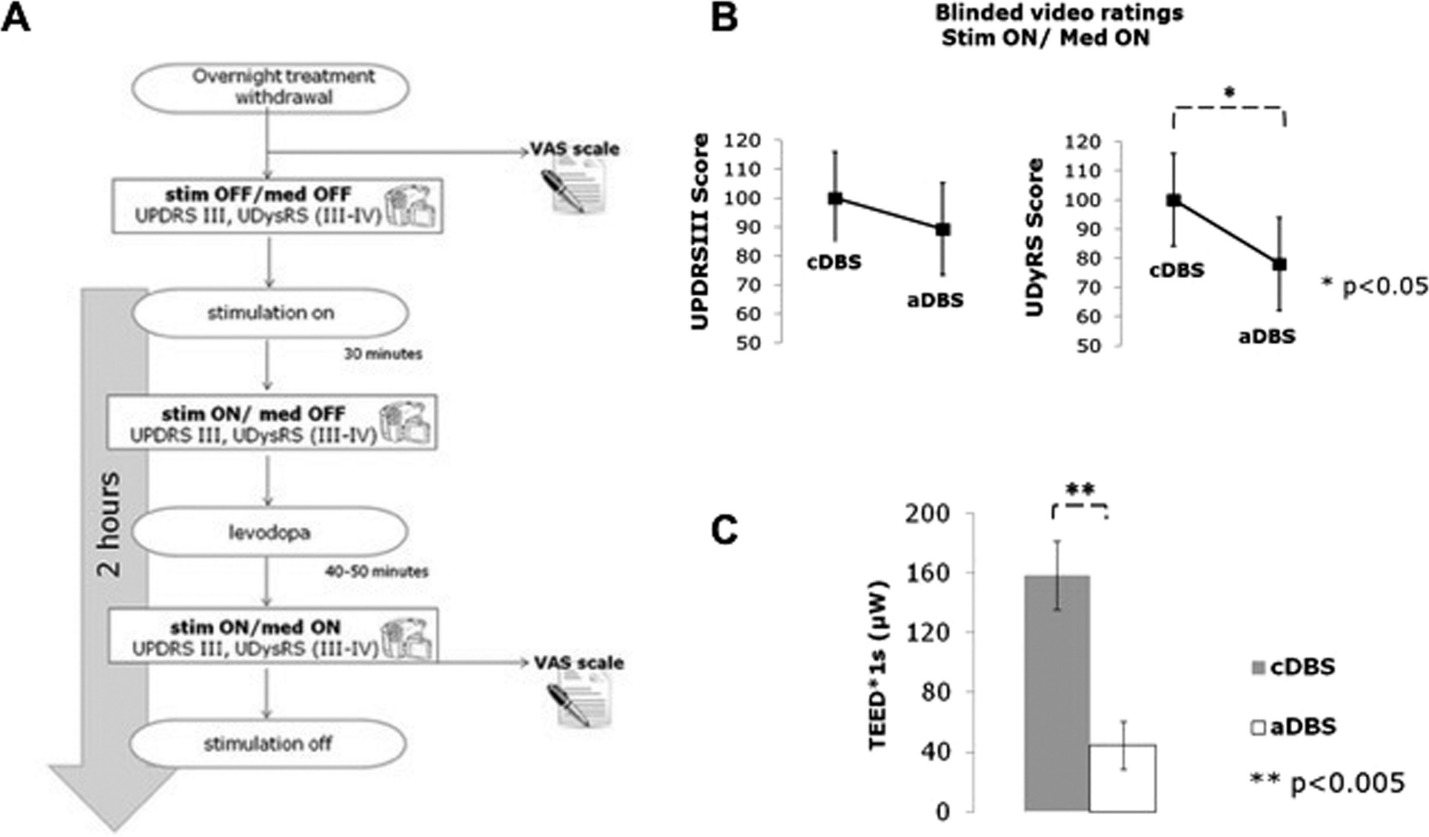
(**A**) Experimental design of each experimental session. Clinical effects were evaluated using the motor part of the Unified PD Rating Scale (UPDRS III) and the Unified Dyskinesia Rating Scale (UDysRS III and IV) during the concurrent administration of DBS (adaptive deep brain stimulation [aDBS] or conventional DBS [cDBS]) and levodopa. (**B**) The UPDRS III and UDysRS scores during aDBS and cDBS, normalized for the maximum score between aDBS and cDBS. (**C**) Total electrical energy delivered (TEED) per unit of time (μW) for aDBS (white color) and cDBS (gray color). Error bars represent the standard error (SE). med, medication; stim, stimulation.

The clinical scores were not significantly different between the 2 experimental sessions at baseline (stimOFF/medOFF UPDRS III, aDBS vs cDBS: 37.0 ± 16.8 vs 36.6 ± 16.2; *F*_1,9_ = 0.2, *P* > .05). When the patient was under the effect of both levodopa and DBS (stimON/medON), we observed a similar improvement on global motor symptoms regardless to the type of DBS (UPDRS III percent change from baseline, aDBS vs cDBS: −46.1% ± 10.5% vs −40.1% ± 17.5%; *F*_1,9_ = 0.6, *P* > .05; Fig. 1B). Conversely, in this condition, aDBS was more effective on dyskinesias than cDBS (UDysRS score, aDBS vs cDBS: 11.7 ± 67 vs 15.0 ± 8.7; *F*_1,9_ = 6.1, *P* = .02; Fig. 1C). These results were obtained with an average power saving of 73.6% ± 22.9% in aDBS compared with cDBS (mean TEED aDBS vs cDBS: 44.6 ± 47.9 μW vs 158.7 ± 69.7 μW; *F*_1,8_ = 30.4, *P* = .0005). Throughout the entire experiment, we did not observe any serious adverse event specifically linked to DBS.

These results support the idea that aDBS, being effective, efficient, and safe, when administered concomitantly to levodopa could help clinicians limit the severity of side effects induced by the transient summation of DBS stimulation and pharmacological therapy. However, the acute experimental setting, characterized by a microlesional effect and by the presence of edema, is a major limitation for the generalizability of our results that need to be confirmed by other studies conducted in a more chronic condition, possibly with implantable devices.


Manuela Rosa, PhD,^1^ Mattia Arlotti, MS,^1,2^ Sara Marceglia, PhD,^1,3^ Filippo Cogiamanian, MD,^4^ Gianluca Ardolino, MD,^4^ Alessio Di Fonzo, PhD,^5^ Leonardo Lopiano, PhD,^6^ Emma Scelzo, MD,^1^ Aristide Merola, MD,^6^ Marco Locatelli, MD,^7^ Paolo M. Rampini, MD,^7^ and Alberto Priori, PhD^1,8^*
^1^Clinical Center for Neurostimulation, Neurotechnology, and Movement Disorders, Fondazione Istituto di Ricovero e Cura a Carattere Scientifico (IRCCS) Ca' Granda Ospedale Maggiore Policlinico, Milan, Italy
^2^Department of Electronics, Computer Science and Systems, University of Bologna, Cesena, Italy
^3^Department of Engineering and Architecture, University of Trieste, Trieste, Italy
^4^Unit of Clinical Neurophysiology, Fondazione IRCCS Ca' Granda Ospedale Maggiore Policlinico, Milan, Italy
^5^Dino Ferrari Center, Neuroscience Section, Fondazione IRCCS Ca' Granda Ospedale Maggiore Policlinico, Milan, Italy
^6^Department of Neuroscience “Rita Levi Montalcini,” University of Turin, Turin, Italy
^7^Unit of Stereotactic Functional and Neuroendoscopic Neurosurgery, Fondazione IRCCS Ca' Granda Ospedale Maggiore Policlinico, Milan, Italy
^8^Department of Health Sciences, University of Milan & Ospedale San Paolo, Milan, Italy

## Author Roles

1) Research project: A. Conception, B. Organization, C. Execution; 2) Statistical Analysis: A. Design, B. Execution, C. Review and Critique; 3) Manuscript: A. Writing of the first draft, B. Review and Critique.

M.R.: 1B, 1C, 2B, 3A

M.A.: 1B, 1C, 2C, 3B

SM: 1B, 2A, 2C, 3A

F.C.: 1B

G.A.: 1B

A.D.F.: 1C

L.L.: 3B

E.S.: 3A

A.M.: 2C

M.L.: 1C

P.M.R.: 1C, 3B

A.P.: 1B

## Full financial disclosures for the previous 12 months

AP and SM were consultant for Newronika srl in the last 12 months
